# Relationship between quality of life and unprotected anal intercourse among Chinese men who have sex with men: a cross-sectional study

**DOI:** 10.1186/s12889-016-3076-z

**Published:** 2016-05-10

**Authors:** Yaxin Zhu, Jie Liu, Bo Qu, Bingxue Hu, Yang Zhang

**Affiliations:** Department of Health Statistics, School of Public Health, China Medical University, No. 77 Puhe Road, Shenyang North New Area, Shenyang, Liaoning Province 110122 People’s Republic of China

**Keywords:** Men who have sex with men, Unprotected anal intercourse, Quality of life, WHOQOL-BREF, HIV/AIDS

## Abstract

**Background:**

The prevalence of unprotected anal intercourse (UAI) is high among Chinese men who have sex with men (MSM). As important aspects of quality of life (QOL), psychological health and social relationships have been found to be associated with UAI among MSM, which was of great concern for intervening on UAI.

**Methods:**

We conducted a cross-sectional study in Zhengzhou, Henan province, and in Huludao, Liaoning province, China, to measure quality of life (QOL) using the brief version of the World Health Organization Quality of Life (WHOQOL-BREF). Cronbach's α coefficient was used to test the internal consistency of the questionnaire items, and construction validity was assessed by exploratory factor analysis. *T*-test, chi-square test and multivariate logistic analysis were carried out to test the relationship between quality of life and unprotected anal intercourse.

**Results:**

The questionnaires of 320 of the 373 men who have sex with men that were surveyed met our validity criteria (response rate: 85.8 %). A total of 161 subjects (50.3 %) reported having unprotected anal sex in the last 6 months. The results of univariate analysis indicated that having unprotected anal sex was significantly associated with psychological health (PSYCH), social relationships (SOCIL), age, and marital status (*P*-value <0.05). The 3 items (“Negative feelings”, “Hopeness on your life”, and “Be able to concentrate”) in the PSYCH subscale were associated with UAI significantly (*P*-value <0.05). Multivariate analysis showed that psychological health (adjusted odd ratio (AOR) 0.979, 95 % confidence interval (CI) 0.961-0.998) was independent factor affecting the likelihood of having UAI in the population of Chinese MSM, and participants who aged 45 or more had higher odds of UAI (AOR 3.986, 95 % CI 1.199-13.255).

**Conclusions:**

WHOQOL-BREF was acceptable for evaluating the quality of life of MSM. Psychological health, as one important aspect of quality of life, was negatively associated with unprotected anal intercourse. The finding underscored the needs to incorporate the strategies on improving psychological health into the UAI intervention to reduce the HIV transmission among MSM.

## Background

HIV epidemics have been declining in many countries and in various populations, yet the incidence is increasing within men who have sex with men (MSM) population in most countries, including China [1–3]. Although the national HIV sentinel surveillance data showed that the HIV-positive rate of risk groups, including injection drug users, female sex workers, and pregnant women, have dropped somewhat or maintained a low level, the rate among Chinese MSM has increased by more than 8 fold from 0.9 % in 2003 to 7.3 % in 2013 [4]. Furthermore, of newly diagnosed cases of HIV/AIDS each year, the percentage of male homosexually transmitted cases has increased from 2.5 % in 2006 to 21.4 % in 2013 [4]. Thus, MSM remains an important driver of Chinese HIV/AIDS epidemic.

Globally, unprotected anal intercourse (UAI) has been indicated as a major risk behavior for HIV infection among MSM [5–9]. Meta-analysis of 82 studies showed that the UAI rate was 53 % among Chinese MSM, and the high rate may result in a serious HIV epidemic [10]. Previous studies have suggested that intervention efforts aimed at reducing UAI may facilitate a reduction in HIV transmission [11, 12]. Such factors, including mental issue [13], substance or alcohol use [14], HIV knowledge [15], social stigma and support [16], have been proven determinants of UAI. Wim *et al.* reported that having a history of mental health problems, such as depressive symptoms, was associated with higher rates of UAI and HIV transmission among MSM [13, 17]. A study on MSM showed that less-supportive social relationship was related to higher rates of unsafe sex [16]. And taking various influencing factors on HIV-risk behavior into account for proposing intervention strategies could produce more sustained reduction in HIV transmission among MSM [7, 18, 19]. Thus, surveying mental health and social roles, which are important aspects of quality of life (QOL) [20], should be of great concern for intervention aimed at reducing the risk of HIV [9].

Quality of life (QOL) is regarded as a good measure of overall health [21]. Previous studies on QOL have been conducted among MSM population, and indicated that surveying the QOL of the MSM can inform us of their physical, psychological, cognitive and social functions according to the individuals’ perceptions their health [22–24]. Dey *et al*. also suggest that the association between QOL and health-risk behavior, including UAI, cigarette smoking, cannabis use, may provide insight into the reasons why a person may engage in these particular behaviors [25]. And Bouhnik *et al.* observed that poor mental health-related QOL was associated with UAI in French HIV-infected gay men [23].

In the present study, the brief version of World Health Organization quality of life (WHOQOL-BREF) was used to assess the QOL. WHOQOL-BREF is an international cross-culturally comparable quality of life assessment instrument to assess the whole health conditions [26], and this scale not only provides a direct quantitative indication of the physical and mental health states, but also provides information about social relationships and environment [27]. There have been several studies on evaluating QOL of MSM using WHOQOL-BREF [20, 28]. The Chinese version of WHOQOL-BREF has proven reliable and valid among MSM population [29, 30].

The aim of the present study was to explore the relationship between QOL and UAI within the Chinese MSM population, and provide the local Center for Disease Control and Prevention (CDC) with some suggestion on UAI intervention to reduce the HIV transmission among MSM.

## Methods

### Ethics statement

This study was approved by the Bioethics Advisory Commission of China Medical University. Written informed consent was obtained from each of the participants.

### Participant recruitment

We conducted a cross-sectional study from April to June of 2014 in Zhengzhou, Henan province and in Huludao, Liaoning province, China. Respondents were recruited from gay bars, gay saunas, and parks. Participants were eligible if they were at least 18 years old and had anal sex with men in the last 6 months before our study. There were 320 valid questionnaires obtained from the 373 MSM surveyed (response rate: 85.8 %).

### Procedures

Eligible MSM completed the questionnaire in a private room using the standardized version on their own. And if they had difficulty in understanding some questions as they filled out the questionnaire, the trained interviewers would provide an explanation. All subjects were given the option to decline participation at any time, and an incentive of 50 Yuan (about 9 USD) was given for participating in the survey.

### Survey questionnaire

Our questionnaire consisted of two parts: a socio-demographic portion and the WHOQOL-BREF. The socio-demographic characteristics included age, marital status, education level, vocation and monthly income. The WHOQOL-BREF includes 24 items in four domains, namely physical health (PHYS), psychological health (PSYCH), social relationships (SOCIL), and environment (ENVIR) and 2 items about overall QOL and general health. The 26 items are scored from 1 to 5, and the scores of four domains are transformed into 0 to 100, with higher scores indicating better QOL. Unprotected anal intercourse (UAI) was defined as having had at least one incident of anal intercourse without a condom with any male partner during the previous 6 months.

### Statistical methods

Cronbach's α coefficient was used to test the internal consistency of the WHOQOL-BREF items, and construction validity was assessed by exploratory factor analysis. *T*-test, *χ*^*2*^ test and multivariate regression analysis were carried out to test the relationship between QOL and UAI rate. All significant variables in the univariate analysis were then entered into a multivariate regression model, and stepwise method was used to select risk factors of UAI in the model. The data were analyzed using SPSS, version 17.0 (SPSS Inc., Chicago, IL, USA) for Windows. A *P*-value <0.05 was considered statistically significant.

## Results

### Socio-demographic characteristics

The median age of this population was 26 years (range 18–63 years). 61 participants (19.0 %) were married. The socioeconomic characteristics of the subjects are shown in Table 1.

**Table 1:** Demographic characteristics of MSM population (*N* = 320)

Variable	Total	UAI		*χ* ^*2*^	*P*-value
		Yes	No		
Age				10.104	0.018
<=25	124 (38.8 %)	57 (46.0 %)	67 (54.0 %)		
26–35	131 (40.9 %)	63 (48.1 %)	68 (51.9 %)		
36–44	43 (13.4 %)	23 (53.5 %)	20 (46.5 %)		
> = 45	22 (6.9 %)	18 (81.8 %)	4 (18.2 %)		
Marital status				4.329	0.037
Single	259 (80.9 %)	123 (47.5 %)	136 (52.5 %)		
Married	61 (19.1 %)	38 (62.3 %)	23 (37.7 %)		
Education				3.062	0.216
Middle school or lower	54 (16.9 %)	33 (61.1 %)	21 (38.9 %)		
Secondary technical school or high school	90 (28.1 %)	44 (48.9 %)	46 (51.1 %)		
College or higher	176 (55.0 %)	84 (47.7 %)	92 (52.3 %)		
Vocation				3.661	0.300
White collar	66 (20.6 %)	31 (47.0 %)	35 (53.0 %)		
Blue collar	217 (67.8 %)	106 (48.8 %)	111 (51.2 %)		
unemployed	19 (5.9 %)	12 (63.2 %)	7 (36.8 %)		
Student	18 (5.6 %)	12 (66.7)	6 (33.3)		
Monthly income				4.805	0.091
<=2000 Yuan	87 (27.2 %)	43 (49.4 %)	44 (50.6 %)		
2001–2999 Yuan	146 (45.6 %)	82 (56.2 %)	64 (43.8 %)		
> = 3000 Yuan	87 (27.2 %)	36 (41.4 %)	51 (58.6 %)		

MSM = men who have sex with men

UAI = unprotected anal intercourse

### Reliability and validity analysis

The overall Cronbach's α coefficient for our analysis was 0.926. The results of Kaiser-Meyer-Olkin (0.917) and the Bartlett's test of sphericity (*χ*^*2*^ = 4033.378, *P*-value <0.001) indicated that the samples in this study are suitable for factor analysis. Exploratory factor analysis showed that the accumulative variance contribution rate was 59.88 %.

### Univariate analysis of factors associated with UAI

A total of 161 subjects (50.3 %) reported having UAI. The univariate analysis (Table 1) indicated that engaging in UAI was statistically associated with older age and being married respectively (*P*-value <0.05).

The PSYCH and SOCIL subscale were found to be inversely associated with practicing UAI (*P*-value <0.05) (Table 2). There are 3 items in the PSYCH subscale, which were “Hopeness on your life”, “Negative feelings”, and “Be able to concentrate”, respectively, were associated with UAI significantly (*P*-value <0.05). The item “Satisfy with your sex life”, which is in the domain of SOCIL, was also significant factor of having UAI (*P*-value <0.05). The results are shown in Table 3.

**Table 2:** The relationship between Quality of life (QOL) and UAI among MSM population by univariate analysis

Domain	UAI		t	*P*-value
	Yes	No		
PHYS (physical health)	67.32 ± 14.69	70.48 ± 13.91	1.942	0.058
PSYCH (psychological health)	59.76 ± 17.09	64.94 ± 14.88	2.891	0.004
SOCIL (social relationships)	63.87 ± 16.67	67.40 ± 14.84	2.000	0.046
ENVIR (environment)	55.57 ± 16.60	57.70 ± 14.00	1.242	0.215
Q1 (general QOL)	3.71 ± 0.87	3.74 ± 0.82	0.295	0.768
Q2 (general health)	3.46 ± 0.88	3.64 ± 0.75	1.853	0.065

MSM = men who have sex with men

UAI = unprotected anal intercourse

**Table 3:** The influence of psychological health and social relationships on UAI among MSM population by univariate analysis

Domain^a^	UAI		t	*P*-value
	Yes	No		
PSYCH (psychological health)				
Hopeness on your life	3.41 ± 1.00	3.69 ± 0.91	2.636	0.009
Negative feelings	3.03 ± 0.89	3.45 ± 0.97	4.067	0.000
Be able to concentrate	3.45 ± 0.94	3.71 ± 0.92	2.539	0.012
SOCIL (social relationships)				
Satisfy with your sex life	3.40 ± 0.82	3.60 ± 0.81	2.267	0.024

MSM = men who have sex with men

UAI = unprotected anal intercourse

^a^Only significant items of PSYCH and SOCIL subscale were showed in the table

### Multivariate analysis of factors associated with UAI

PSYCH score was significant factor affecting UAI in the multivariate logistic regression model (AOR 0.979, 95 % CI 0.961-0.998). Compared with those participants who aged less than 45, the participants aged 45 or more had significant higher odds of engaging in UAI (AOR 3.986, 95 % CI 1.199-13.255). The results are shown in Table 4.

**Table 4:** Multivariate logistic regression models showing risk factors of UAI^a^

Factor	COR (95 % CI)^b^	AOR (95 % CI)^c^
Sociodemographics		
Age		
<=25	1	1
26–35	1.089 (0.666–1.781)	0.998 (0.601–1.658)
36–44	1.352 (0.674–2.710)	1.050 (0.486–2.267)
> = 45	5.289 (1.692–16.531)*	3.986 (1.199–13.255)*
Marital status		
Single	1	1
Married	1.827 (1.031–3.238)*	1.492 (0.774–2.877)
*QOL*		
PSYCH (psychological health)	0.980 (0.966–0.994)*	0.979 (0.961–0.998)*
SOCIL (social relationships)	0.986 (0.972–1.000)*	1.003 (0.984–1.022)

^a^Only factors significant in univariate analysis were included in the multivariate logistic regression model

^b^Univariate logistic regression model

^c^Multivariate logistic regression model

*p <0.05

*COR* crude odds ratio, *AOR* adjusted odds ratio, *CI* confidence interval

## Discussion

50.3 % of subjects reported having UAI. This result is comparable to the reported rates of UAI in Chinese MSM (53 %, 95 % CI: 51 to 56 %) and in MSM in 21 US cities (54 %) [10, 31]. Our study found that psychological health and social relationships as two out of four domains of QOL were statistically significant factors of UAI among MSM in our study. The reliability and validity of the WHOQOL-BREF were acceptable*.* Univariate analysis showed that lower PSYCH and SOCIL subscale scores were significantly related to UAI (*P*-value <0.05). And the older MSM and being married were associated with UAI respectively (*P*-value <0.05). Multivariate analysis showed that PSYCH (AOR = 0.979; 95 % CI: 0.961 to 0.998) and age 45 or more (AOR 3.986, 95 % CI 1.199-13.255) were independent factors influencing UAI in Chinese MSM (*P*-value <0.05).

In line with other studies, we found that poor psychological health was significantly associated with engaging in UAI (P-value <0.05) [9, 23]. More specifically, the lower scores of the items “Negative feelings”, “Hopeness on your life”, and “Be able to concentrate” were determinants of UAI, respectively (*P*-value <0.05). Due to experiencing discrimination about sexual orientation by family and other institutions, many Chinese MSM had encountered stigma, which led to mood, anxiety disorders and depression more common in MSM than in men in the general population [32, 33]. Moreover, increasing evidence has shown that compared to heterosexuals, gays exhibited elevations in hopelessness caused by the stressors that sexual minorities confront [34–36]. Safren *et al.* also indicated that mental health problems may compromise the impact of HIV prevention programs [37]. Behavioral interventions combined with mental health treatment have been shown to increase the effectiveness of reducing UAI and HIV prevention programs among MSM and HIV-infected individuals [37–39]. And previous studies implied that community-based or structural interventions that reduce sexual minority prejudices, in combination with individual-based interventions that address negative beliefs about life, may help to alleviate the high rates of mental health problems among MSM [34, 37]. Our results suggest that improving psychological health is an aspect that should be incorporated into UAI reduction-intervention strategies to facilitate HIV prevention among Chinese MSM.

Our study also showed that weaker social relationships were associated with a higher likelihood of practicing UAI in the MSM population (*P*-value <0.05). Given homosexuality is generally viewed as unacceptable in Chinese culture, MSM tend to report more social isolation, and avoid entering close relationships for fear of others’ discovering their sex orientation, which led this population to limited access to the public health care system aimed at gay community [34, 40, 41]. Previous study also suggested that sexual risk prevention interventions should take addressing their capacity to build adequate and supportive social relationships into account to enhance HIV prevention programs among MSM population [16]. Furthermore, we also found that lower score of the “Satisfy with your sex life” item which included in the SOCIL domain was significantly related to engaging UAI. Tsui *et al.* also found that lack of sexual pleasure was significantly associated with UAI among MSM in Shenzhen and HongKong [42]. A study on HIV-positive MSM showed that sex toy users reported higher levels of sexual satisfaction, and suggested that the effect of sex toys as tools on enhancing sexual pleasure and reducing HIV transmission should be explored in further studies [43].

Our results also found that older Chinese MSM were more likely to engage in UAI than younger MSM, which is consistent with several other studies of Chinese MSM [44, 45]. In addition, according to the national report on HIV/AIDS cases, new HIV cases among the higher age group increased noticeably [4, 46]. There could be several reasons to explain why the UAI rate of Chinese older MSM was higher. Firstly, few HIV intervention efforts aimed at reducing UAI have focused on older MSM, for the inaccurate belief that older men are not sexually active [45]. Secondly, ageism and homosexual stigma could cause older MSM to hide their homosexual activity, thus increasing the difficulty of reaching them by prevention intervention [47, 48]. Thus, targeted interventions on reducing UAI in this HIV-risk subgroup could be of great need.

In sum, UAI intervention programs targeting MSM need to be evidence-based and would benefit from sound research support. Our study also had some limitations. Firstly, random sampling was not performed, and the sampling was venue based in this study. Secondly, the study was conducted in only two Chinese cities, and the results may not be representative of all, or even most, Chinese MSM. And in our study, the questionnaire in which more than 20 % of items were left unanswered was considered to be invalid. 15 % of the respondents only completed below 80 % of the questionnaires, probably because they may be not willing to expose their personal information, such as age, sexual behavior. This may be why nearly 15 % of surveys did not meet the criteria of "valid". Further studies in more areas of China will determine whether these findings can be generalized.

## Conclusions

Poorer psychological health and social relationships, as the important aspects of QOL, were found in the present study to be associated with higher likelihood of engaging in UAI, and psychological health played a larger role. It therefore appears necessary to incorporate improved psychological health into future intervention strategies to prevent UAI in MSM.

### Ethical approval and consent to participate

This study was approved by the Bioethics Advisory Commission of China Medical University. Written informed consent was obtained from each of the participants.

### Consent to publish

Not applicable.

### Availability of data and materials

All data is available in the paper. Data will be shared upon request and it is subjected to the data protection regulations.

## Abbreviations

UAI: unprotected anal intercourse
QOL: quality of life
MSM: men who have sex with men
PSYCH: psychological health
PHYS: physical health
PSYCH: psychological health
SOCIL: social relationships
ENVIR: environment
